# Development and Degradation Study of PLA‐Based Medical Implant Markers for Magnetic Particle Imaging

**DOI:** 10.1002/mabi.202400495

**Published:** 2025-02-06

**Authors:** Patrick N. Elfers, Kerstin Lüdtke‐Buzug, Ankit Malhotra, Justin Ackers, Liana Mirzojan, Maximilian Wattenberg, Johann C. Engster, David Melenberg, Mandy Ahlborg, Thomas Friedrich, Maria‐Josephina Buhné, Malte M. Sieren, Thorsten M. Buzug, Roman Kloeckner, Jörg Barkhausen, Franz Wegner

**Affiliations:** ^1^ Institute for Interventional Radiology University Hospital Schleswig‐Holstein Ratzeburger Allee 160 23538 Lübeck Germany; ^2^ Fraunhofer IMTE Fraunhofer Research Institution for Individualized and Cell‐Based Medical Engineering Mönkhofer Weg 239a 23562 Lübeck Germany; ^3^ Institute of Medical Engineering University of Lübeck Building 64, Ratzeburger Allee 160 23562 Lübeck Germany; ^4^ Institute of Radiology and Nuclear Medicine University Hospital Schleswig‐Holstein Ratzeburger Allee 160 23538 Lübeck Germany

**Keywords:** magnetic particle imaging, medical instruments, nanoparticles, PLA degradation

## Abstract

Magnetic particle imaging (MPI) is a promising imaging modality nearing clinical introduction. MPI's tracer‐based principle allows for highly sensitive background‐free imaging. Potential clinical applications include cardiovascular imaging and endovascular interventions. In principle, medical instruments are invisible in MPI due to the missing signal generation. Therefore, permanent marking technologies have been introduced. Additionally, temporary markers are of interest for follow‐up examinations after stent implantation to prevent artifacts during postinterventional stent lumen quantification. Consequently, medical instrument markers for MPI, based on biodegradable polylactic acid (PLA) and superparamagnetic iron‐oxide nanoparticles (SPIONs), are developed in this study. To investigate the markers, signal characteristics and degradation over time are studied for 28 d in a water bath at 37 °C. The samples are analyzed using a scale, micro‐CT, microscopy, magnetic particle spectroscopy (MPS), MPI, and vibrating sample magnetometry (VSM). A continuous mass decrease is detected (≈90% after 28 d), while MPS and MPI data show no loss of signal. VSM confirms that the markers’ mass reduction can be accounted for the degradation of PLA, while the SPIONs hardly detach from the coating. The introduced marking technology, with its degradation characteristics and signal behavior, is the basis for a variety of anticipated medical application scenarios.

## Graphical abstract

Nonpermanent marking technology is needed for medical instruments for accurate visualization in magnetic particle imaging. In this work, markers based on superparamagnetic iron‐oxide nanoparticles (SPIONs) and polylactic acid (PLA) are developed and investigated over a time frame of 28 days. Their degradation and signal characteristics provide the basis for a variety of clinical application scenarios. Created in BioRender. Klöckner, R. (2025) https://BioRender.com/ l02b084

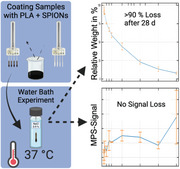

## 1 Introduction

Magnetic particle imaging (MPI) is an emerging tomographic technique nearing clinical introduction.^[^ ^1^, ^2^ ^]^ The main principle is the visualization of the spatial distribution of superparamagnetic iron‐oxide nanoparticles (SPIONs) by static and oscillating magnetic fields.^[^ ^3^ ^]^ MPI offers high temporal and spatial resolution as well as real‐time imaging capabilities. In the last decade, several fields of application were identified and studied intensively. One of the most promising use cases of MPI is cardiovascular imaging and the guidance of endovascular interventions.^[^ ^4^, ^5^, ^6^, ^7^ ^]^ For this use case, the lack of ionizing radiation is especially beneficial for patients as well as medical staff. Additionally, MPI can quantify vascular stenoses and stent lumina very accurately.^[^ ^8^ ^]^ Due to MPI's tracer‐based principle, most of the so far tested commercially available medical instruments and implants are invisible in MPI.^[^ ^8^, ^9^ ^]^ Thus, dedicated marking technologies became necessary. Here, multiple varnish‐based techniques have been reported.^[^ ^10^, ^11^, ^12^ ^]^ These introduced markers are typically permanent and may interfere with subsequent SPION‐based imaging. A concrete application scenario is MPI‐guided stent implantations. For the accurate placement of a stent – an endovascular implant – markers at the stent tips are essential. However, for potential follow‐up examinations, the accuracy might be influenced negatively by a permanent SPION marker. Therefore, a degradable marker is needed for the long‐term perspective of non‐invasive stent imaging. Furthermore, degradable stents are available to prevent in‐stent stenoses.^[^ ^13^ ^]^ In this regard, controlled degradation of both the stent itself and the markers is beneficial.

The so far presented varnishes need additional coatings to guarantee their safe usage, as they are limited regarding their biocompatibility.^[^ ^11^ ^]^ In consequence, new materials based on the combination of medical polymers and SPIONs have been introduced in recent years, offering a broad range of applications. For example, our group showed the possibility to dynamically visualize a balloon catheter based on polymer‐integrated SPIONs with MPI.^[^ ^14^ ^]^ Additionally, the integration of SPIONs in Polylactic acid (PLA) was reported. Jacobi et al. investigated the characteristics of a PLA‐SPION mixture for MPI and could prove its eligibility for imaging.^[^ ^15^ ^]^ Furthermore, 3D printing with PLA‐iron material was tested for MPI.^[^ ^16^ ^]^ In contrast to varnishes, PLA is biocompatible and degradable.^[^ ^17^ ^]^ Thus, the use of a PLA‐SPION mixture is advantageous for the development of non‐permanent markers for MPI.

This study reports the development of implant markers for MPI based on a PLA‐SPION mixture. It investigates the imaging and degradation characteristics over a time frame of 4 weeks.

## 2 Experimental Section

### 2.1 PLA Synthesis

The primary objective in developing a synthesis method for PLA was to obtain optimal viscosity to ensure good coatability. This is essential for producing thin, uniform coatings on devices. Additionally, achieving thermostable samples at 37 °C was a critical goal. To achieve these objectives, the synthesis parameters were determined iteratively.

Based on Jacobi et al.,^[^ ^15^ ^]^ 80–100 mg of SnCl_2_ (Tin(II) chloride dihydrate, Carl Roth, Karlsruhe, Germany) were added to 10 mL of lactic acid (DL‐Lactic acid, Carl Roth, Karlsruhe, Germany) inside a 25 mL beaker for synthesizing PLA. A magnetic stirring bar was added. The beaker was put into a silicone oil bath on a heating plate with an integrated magnetic stirrer (RCT Basic, IKA‐Werke, Staufen, Germany). The temperature was controlled with a digital thermometer (IKATRON ETS‐D4 fuzzy, IKA‐Werke, Staufen, Germany) connected to the heating plate. The mixture was stirred for 5 min before being heated up to 80 °C, which was maintained for 15 min to obtain a homogenous reaction mixture. The temperature was then increased to the reaction temperature of 160 °C.^[^ ^18^ ^]^ The duration of how long the reaction temperature applied to the lactic acid is crucial for the extent of the polycondensation process of the lactic acid. To assess the dependence of the final properties of the PLA on the synthesis duration, the synthesis was carried out for different time periods at 160 °C (1 h 30 min, 1 h 50 min, 2 h 10 min, 2 h 30 min).

After the time elapsed, the beaker was taken out of the oil bath, the stirring bar was removed and SPIONs (Iron(II, III) oxide nanopowder, Sigma–Aldrich, Merck, Darmstadt, Germany) were added to the PLA within 30 s. The mixture was stirred with a self‐built electrical stirrer for 45 s to ensure a homogenous distribution of the particles.

**Figure 1:**
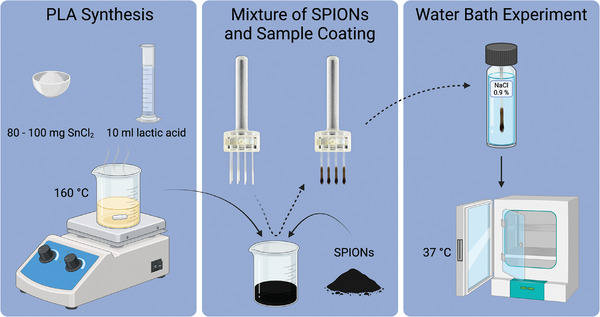
First, PLA was synthesized by heating lactic acid with a catalyst inside an oil bath (the oil bath is not depicted for simplicity). Next, the beaker with the PLA was taken out of the oil bath, and SPIONs were added and mixed to distribute them homogeneously. Lastly, the glass capillaries were coated by a dip coating process using a 3D‐printed sample mount. The final samples were then exposed to the water bath inside an incubator. Created in BioRender. Klöckner, R. (2025) https://BioRender.com/ l02b084.

To exclude any magnetic interferences during the measurements, glass capillaries (Microcaps, 2 µL, Drummond Scientific Company, Broomall, PA, USA) instead of metallic stent struts were coated by dipping them into the PLA‐SPION mixture. To generate homogenous samples, a specific mount was designed and 3D‐printed to hold up to 16 glass capillaries during a dip coating procedure (**Figure** **1**). Three sets of 16 capillaries each were dip‐coated with a different waiting time (1:30 min, 3:00 min, and 5:00 min) after taking the beaker out of the oil bath. The capillaries were immersed in the PLA‐SPION mixture until they contacted the bottom of the beaker to ensure a consistent depth during the dip coating process. They were held in place for 1 s and then removed, dried, weighed, and placed in a drying cabinet (BM 500, Memmert, Schwabach, Germany) set to 37 °C to investigate their thermostability.

After every experiment, varying the duration of the synthesis and the timing of coating the samples, the different characteristics of the PLA, e.g., viscosity, coatability, and temperature stability at 37 °C, were evaluated visually. Maintaining 160 °C for 1:50 h and conducting coating for 3 min after taking the beaker out of the oil bath delivered the lightest stable coatings. Consequently, these parameters were used for the following experiments of this work. If heated longer, the viscosity of the PLA increased, which made it difficult to coat the capillaries and generated heavier coatings. If heated shorter, the PLA was not stable enough to maintain its shape when exposed to 37 °C.

### 2.2 Concentration Series

To determine the ideal concentration of SPIONs in the PLA‐SPION mixture for imaging, a concentration series was performed. For this purpose, the PLA was synthesized as described before. Instead of adding the SPIONs directly into the 25 mL beaker after the synthesis, the PLA was pipetted into five different glass tubes (Rotilabo Test Tubes, 4 mL, Carl Roth, Karlsruhe, Germany). Each glass tube was weighed empty, preheated to 160 °C in a separate oil bath, and filled with ≈1 mL of PLA. The glass tubes were then cooled down and weighed again to determine the actual weight of the PLA in each tube. This was necessary as with dropping temperatures, PLA hardens, which made it particularly challenging to pipette PLA. The pipette tips (MultiFit Pipette Tips, 100–1000 µl, Sorenson Bioscience, Murray, UT, USA) were therefore preheated to 120 °C (maximum tolerated) to ensure feasibility. Preheating was performed in a drying cabinet (T 5042 EK, Heraeus, Hanau, Germany).

**Figure 2:**
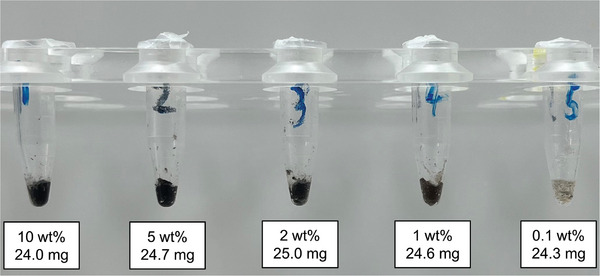
Concentration series of PLA‐SPION mixture in Eppendorf tubes, with the mass fraction (wt%) of the SPIONs (top text) and the filling mass (bottom text).

In the next step, freeze‐dried SPIONs (Perimag, micromod, Rostock, Germany) were weighed into the PLA‐filled glass tubes accordingly to generate a series with mass fractions (wt%) of 10%, 5%, 2%, and 1%. In contrast to the preliminary experiments, these SPIONs dedicated to MPI were used for better performance. Each glass tube was then inserted back into the oil bath to induce the liquid state and the contents were stirred with the self‐built electrical stirrer to generate a homogenous PLA‐SPION mixture. The tubes were then taken out of the oil bath to let the mixture cool down and harden. Afterward, a bone marrow biopsy system (Bone Marrow, SOMATEX Medical Technologies, Berlin, Germany) was used to remove a small cylindrical sample of each PLA‐SPION mixture. These were filled into a 0.5 mL Eppendorf tube (**Figure** **2**). One Eppendorf tube was filled with pure SPIONs for comparison. The MPI signal characteristics of all samples were then measured in a Magnetic Particle Spectrometer (MPS, see below).^[^ ^19^ ^]^

### 2.3 Water Bath Experiment

To investigate the degradation characteristics of the optimized PLA‐SPION mixture, an experiment was conducted with 30 samples divided into ten groups with three samples each. Every group was incubated (CB 160, Binder, Germany) at 37 °C for a different duration (1 h, 2 h, 6 h, 24 h, 48 h, 72 h, 7 d, 14 d, 21 d, 28 d).

To generate samples for the water bath experiment, the PLA was synthesized as already described. After the synthesis, the beaker was taken out of the oil bath, and within 30 s, 148 mg of SPIONs (freeze‐dried Perimag, micromod, Rostock, Germany) were added to the PLA, which resulted in ≈2 wt% of SPIONs in the product. The weight of the PLA inside the beaker before adding the SPIONs was approximated beforehand by repetitive synthesis experiments. With these, it could be estimated that the PLA inside the beaker was 7.26 g at the end of the synthesis. The PLA‐SPION mixture was then stirred for 45 s to distribute the SPIONs homogenously inside the PLA.

Three loaded sample holders were used to generate 48 samples in total. The capillaries were coated as described before, this time sequentially at 2:50, 3:10, and 3:30 min after taking out the beaker to coat the capillaries as close to the optimal 3 min as feasible.

The samples were dried, weighed, measured in the 1D‐MPS, and scanned in the micro‐CT before and after the water bath. 30 samples were chosen with a mean coating mass of 8.3 mg ± 0.46 mg. Each sample was put into a 25 mL glass beaker filled with 22 mL of isotonic saline solution. The capillaries were placed upside down and held in place with foam and plastic columns to ensure they did not touch the wall of the glass beaker. The beakers were sealed with a lid. All samples were inserted into the glass beakers within 5 min and placed in the incubator set to 37 °C right after. At their respective time points, the beakers were removed from the incubator. The coated capillaries were retrieved from the water bath and placed inside a lidded box filled with silica gel (Silica Gel Orange, Carl Roth, Karlsruhe, Germany) for 48 h to completely dry them before analysis.

### 2.4 Micro‐CT Scans

To analyze the degradation process inside the coating, the samples were scanned with an FF35 CT system (Comet Yxlon GmbH, Hamburg, Germany). The samples were scanned with a tube voltage of 110 kV and a tube current of 540 mA. The set magnification of 14.8, combined with a detector binning of two and a pixel pitch of 150 µm, resulted in a tomogram resolution of 20.3 µm. The tomogram was reconstructed using an FDK‐filtered back‐projection approach and auto‐alignment of the projection images. Reconstruction was performed using CERA (Siemens Healthineers AG, Erlangen, Germany).

### 2.5 Microscopy

Microscopy of the samples was performed after the water bath using the SteREO Discovery.V8 (Carl Zeiss Microscopy, Oberkochen, Germany). Additionally, a reference sample not exposed to the water bath was examined to represent the state before exposure.

### 2.6 MPS Measurements

To characterize the MPI signal of the PLA‐SPION mixture, MPS measurements were performed in a 1D‐MPS device^[^ ^19^ ^]^ using 0.5 ml Eppendorf Tubes as sample holders. The concentration series as described above could be measured directly, while the glass capillaries had to be shortened in length after being weighed to fit them into the Eppendorf Tubes. The samples were weighed again after being shortened.

The samples dynamic magnetization responses were acquired under an applied sinusoidal excitation field at 25 kHz with an amplitude of 20 mT. The received signal was averaged for 0.4 s, and subsequently, background and transfer functions were corrected. To ensure comparability between the different samples, all measurements were normalized to the respective SPION weight as well as the total sample weight (PLA+SPIONs).

### 2.7 MPI Scan and Image Reconstruction

The MPI scans were conducted after the water bath with the aforementioned reference sample representing the state before exposure. The Bruker preclinical MPI system (MPI 25/20FF, Bruker BioSpin, Ettlingen, Germany) was used with a selection field gradient setting of 2 T m^−1^ and 3D Lissajous excitation with 12 mT excitation fields in all three dimensions. For the system matrix calibration, a robot‐based calibration scan was acquired using a 2 × 2 × 2 mm^3^ sample of Perimag‐loaded PLA with a discretization of 36 × 20 × 8 positions spanning a calibration‐FOV of 36 × 20 × 11 mm^3^ which was chosen to match the elongated sample geometry and differs in the aspect ratio compared to the excitation field of view of 24 × 24 × 12 mm^3^.

For reconstruction, the regularized least‐squares problem was solved using the implementation of Kaczmarz’ algorithm with Tikhonov regularization (*λ* = 10^−4^) of the software package MPIReco.jl.^[^ ^20^ ^]^ Only frequencies with a signal‐to‐noise ratio above 5 were used for reconstruction and all frequency components were weighted to achieve whitening of the system noise.^[^ ^21^ ^]^ The reconstruction parameters are identical for all shown reconstructions.

### 2.8 Vibrating Sample Magnetometer (VSM) Measurements

Two of the above‐mentioned reference samples and two samples, which were exposed to the water bath for 28 d, were analyzed in a VSM (Lakeshore 8600 VSM) to measure the saturation magnetization of the samples at an applied external magnetic field of 2 T. Assuming no change in the oxidation state of the magnetic particles, the saturation magnetization should directly correlate to the total amount of magnetic nanoparticles in the sample and unlike MPS will not be affected by their binding state. As in the MPS measurements, the magnetic moment measurements were normalized to the total mass of the sample before the water bath, including both PLA and SPIONs.

## 3 Results

### 3.1 Concentration Series

**Figure 3:**
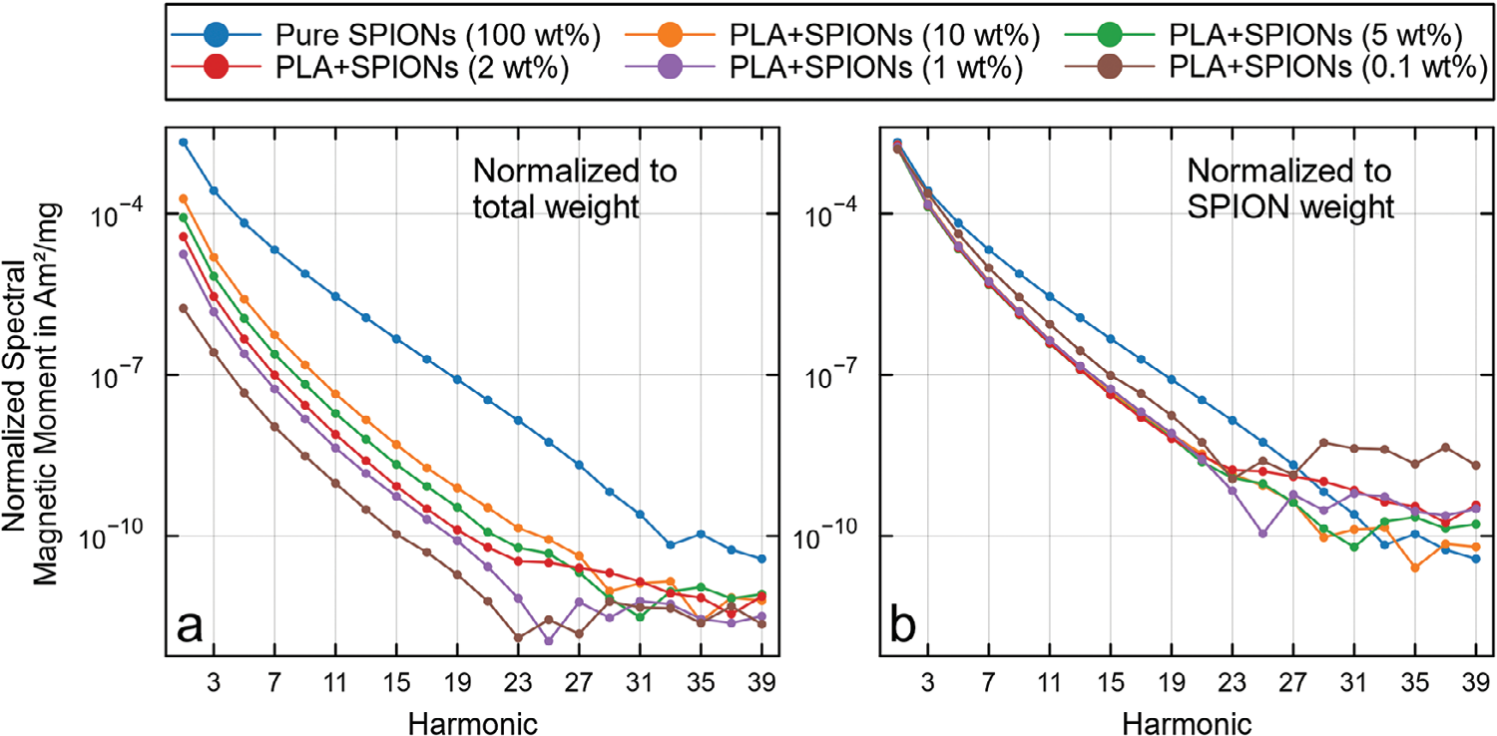
a,b) 1D MPS amplitude spectra of different SPION concentrations in the PLA‐SPION mixture normalized to the total sample weight (PLA+SPIONs) and to the SPION weight. Only the odd harmonics are shown. As expected, the signal increases with the SPION content, as seen in the vertical offsets (a). The addition of PLA causes a signal decrease in all PLA‐SPION samples up to the 27th harmonic (b).

All five samples generated a detectable MPS signal with a positive correlation between nanoparticle concentration and signal intensity (**Figure** **3**). The signal shape does not change between the different concentrations. However, the decay toward the higher harmonics of the PLA‐SPION mixture is slightly faster compared to the signal of the pure nanoparticles, indicating a slight loss in MPI performance due to the mixing.

### 3.2 Water Bath Experiment

**Figure 4:**
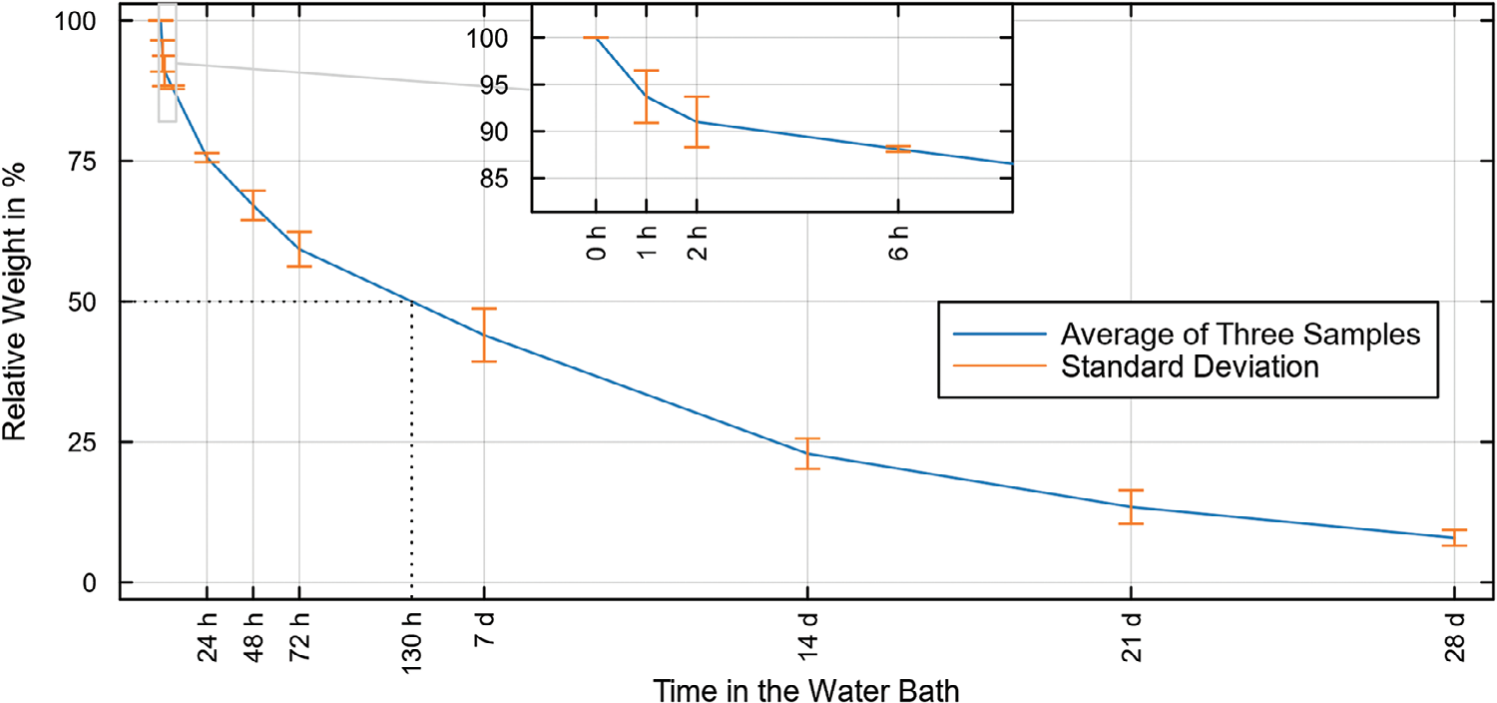
Mass loss of the PLA‐SPION samples over a time frame of 28 d in the water bath.

The degradation experiment in the water bath revealed a continuous decrease in weight with ongoing exposure to the water bath. After 7 d, an average of 56% weight reduction could be seen (**Figure** **4**). After 28 d, an average of 92.1% of the coating was degraded. The approximated decay half‐life extracted from the plot was 130.3 h (5.4 d).

### 3.3 Micro‐CT Scans and Microscopy

**Figure 5:**
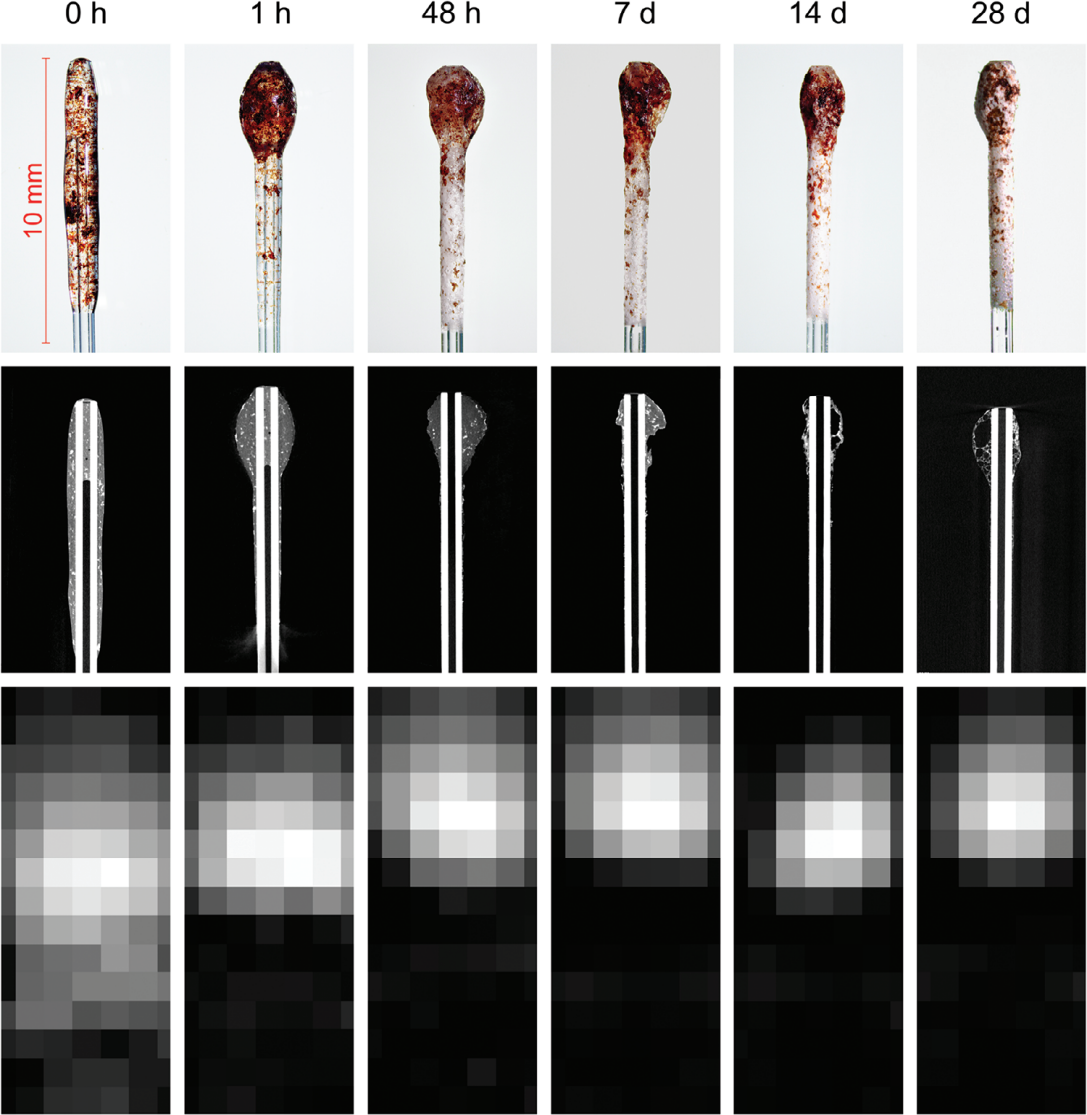
Microscopic images (top), micro‐CT images (middle), and sections of the MPI reconstructions (bottom) of exemplary samples not exposed to the water bath and after their respective durations of exposition to the water bath. An arbitrary slice of the interesting section was chosen for the micro‐CT images. The gray scale in the MPI reconstructions is adjusted to every reconstruction separately. All images have the same scaling.

Microscopic images showed the initially transparent coating with evenly distributed SPIONs (**Figure** **5**). Within the first hour of exposure to the water bath, the PLA‐SPION mixture concentrated at the tip of the sample, while particle dislocation was rather marginal in the following time points. Additionally, a white layer formed on the surface of the samples with prolonged exposure, reducing the coating's transparency. This phenomenon was first observed after 48 h and became distinct on the seventh day. This layer was later identified as sodium chloride crystals by a flame test.

Micro‐CT measurements revealed the formation of cavities inside the coating during degradation, accounting for the mass reduction while maintaining the external shape (Figure 5).

### 3.4 MPS Measurements of the Water Bath Samples

**Figure 6:**
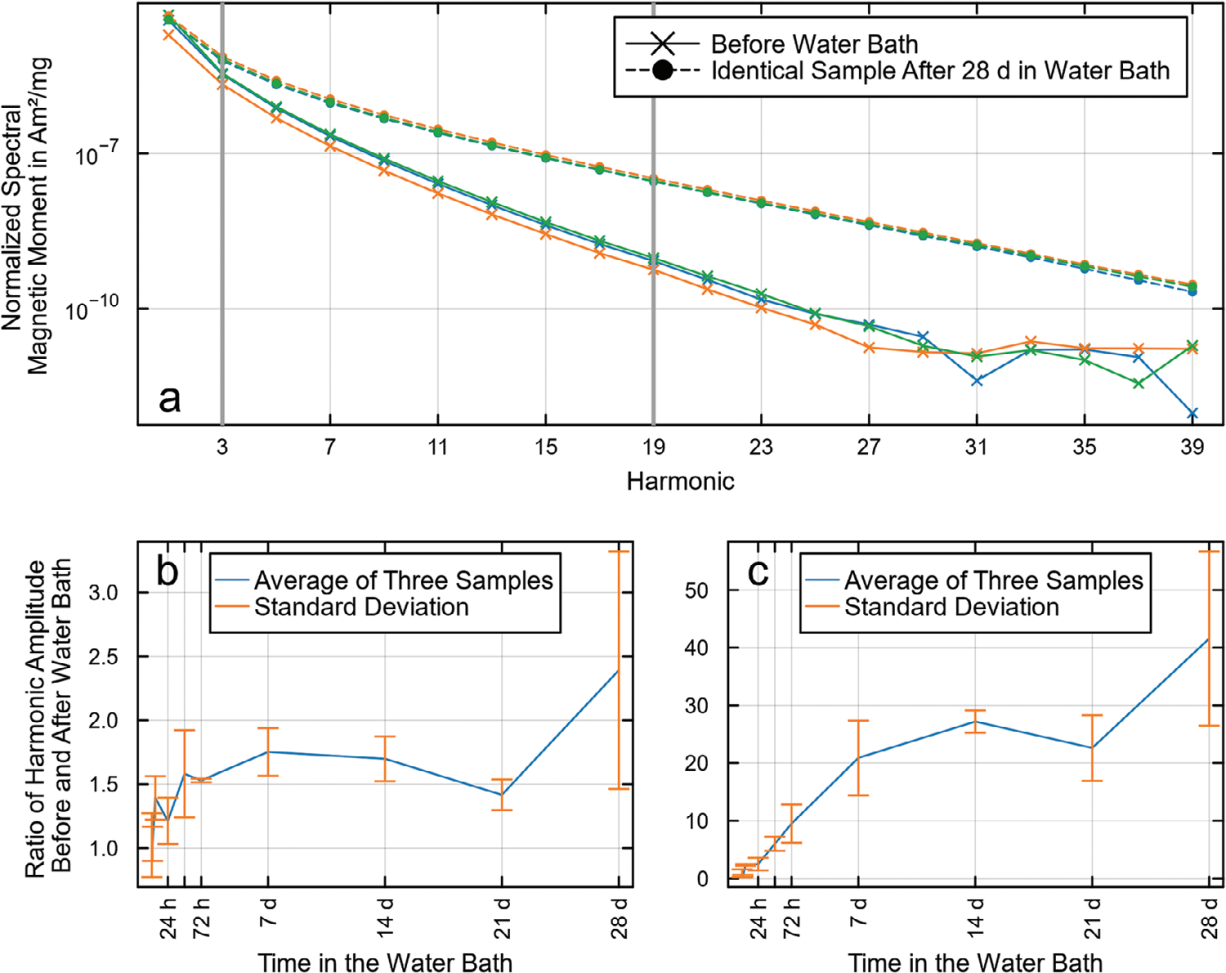
a) 1D‐MPS measurements of three samples before and after 28 d water bath exposure show a signal increase after the water bath. Each sample was normalized to its weight before the water bath. b,c) The amplitude ratio before and after the water bath showed only a slight increase for the third harmonic, while a clear increasing trend is visible for the 19th harmonic.

Even though the mass of the coating decreased by over 90% after 28 d, the 1D‐MPS measurements showed an increase in signal intensity, especially of the higher harmonics (**Figure** **6**a). Comparing the ratio of the MPS signal of identical samples before and after the water bath showed the same trend. While the third harmonic showed only a slight increase with increased time in the water bath (Figure 6b), the 19^th^ harmonic increased up to 50‐fold following 28 d in the water bath (Figure 6c). The signal seemed to increase linearly for the first week in the water bath and thereafter it evolves toward a saturation of around a 30‐fold signal increase.

### 3.5 VSM Measurements

To validate if the total amount of magnetic material has been reduced during the exposure to the water bath, the saturation magnetization of the samples was measured. The saturation magnetization normalized to the initial coating weight was measured to be on average 0.285 Am^2^ kg^−1^ before the water bath and 0.284 Am^2^ kg^−1^ after the water bath. This leads to the conclusion that the total amount of magnetic particles did not reduce significantly, and most particles were still present inside the remains of the coating.

### 3.6 MPI Measurements and Image Reconstruction

The reconstructed MPI images showed detectable circular‐shaped signal blobs for all samples (Figure 5). The reference sample which was not exposed to the water bath was depicted along its entire coated length. At the tip, there was a slightly stronger signal to observe. With ongoing exposition to the water bath, the centers of signal intensity slightly displaced toward the samples’ tips. Furthermore, the signal blobs showed an increase in sharpness at their boundaries.

## 4 Discussion

In this work, we introduce PLA‐based medical implant markers for MPI. The developed markers showed a significant loss of mass during a period of 4 weeks without a relevant decrease in the MPI signal.

The so far introduced marking technologies for instrument visualization in MPI aimed for permanent markings.^[^ ^4^, ^10^, ^11^, ^12^, ^14^ ^]^ However, there are also application scenarios that need a time‐limited marking approach. In addition to endovascular instruments like stents, there is a variety of scenarios that also could benefit from the introduced degradable SPION‐marking technology in a functional manner., e.g., changes in pH, which could be present due to inflammation, could influence the degradation behavior of the marker and thus add diagnostic value.^[^ ^22^ ^]^ The changes in viscosity during the degradation process will also affect the MPI signal properties of the SPIONs in the PLA and thus can be used for the monitoring of the marker and its biological environment.^[^ ^23^ ^]^ Furthermore, the introduced approach is transferable to magnetic resonance imaging (MRI). Here, SPION‐based instrument markers have been introduced to achieve sufficient visibility of endovascular instruments during MRI‐guided interventions.^[^ ^24^, ^25^ ^]^ The iron content of the SPION‐markers causes susceptibility artifacts that ensure the localization of the instruments during MRI procedures.

In comparison to existing literature, we observed a relatively fast PLA degradation in our work.^[^ ^17^ ^]^ The degradation of PLA depends on its intrinsic properties, which can vary significantly, as well as environmental conditions, making direct comparisons challenging.^[^ ^26^ ^]^ To our knowledge, no MPI signal degradation experiments have been performed with PLA‐SPION mixtures so far. Nevertheless, the observed degradation time could be explained by the mixture of SPIONs into the PLA, as the principle of nanoparticle integration is an established method to steer/increase the degradation rate of PLA.^[^ ^27^ ^]^ The development of cavities in the PLA, which we observed in the micro‐CT images during the degradation, is also a known phenomenon from past studies.^[^ ^28^ ^]^ The influence of the surrounding medium is not expected to influence degradation characteristics and degradation rate up to a timeframe of 12 weeks.^[^ ^29^ ^]^

Unexpectedly, we observed no decrease in particle response, despite a total mass loss of more than 90% in 4 weeks. The deformation of the samples during the water bath may be one explanation for this phenomenon. A softening of the PLA due to water absorption leads to a change in PLA viscosity. On the one hand, this can cause a change in particle response and an increase in the MPI signal, respectively.^[^ ^23^ ^]^ On the other hand, the concentration of SPIONs at the tip of the capillaries might increase due to the PLA displacement under the influence of gravity. Furthermore, both effects could influence the particle‐particle interactions.^[^ ^30^ ^]^ Thus, the increase in MPS signal seems to be only affected by the local environment of the particles, e.g., their binding state or viscosity. The resulting increasing MPI signal is very beneficial for an implanted marker, as the observed signal trend allows for reliable marker monitoring. Only a very tight time interval representing the complete disappearance of the marker is expected.

This in vitro study has several limitations. The degradation experiments have been performed under static conditions. Thus, the evaluation of the degradation and the particle response under flow conditions should be studied in the future. Especially, a longer time interval of testing the samples until they are completely inapparent would be helpful to fully assess the long‐term perspective. To determine the degradation constant of the used PLA‐SPION mixture, it is pivotal that such a specific sample undergoes a long‐term study with different SPION‐PLA mixing ratios, as the decay constant heavily depends upon the constitution of PLA and SPION. Additionally, the environment and the temperature conditions of the samples play a critical role in the degradation process and thus should be intensively studied in future work. Furthermore, we only investigated the combination of a single SPION‐type in one concentration with PLA. Here, the effects of mixing different MPI‐visible SPIONs in varying concentrations with PLA could gain more insights. Besides that, the markers need further investigation and optimization regarding mechanical stability. The deformation of the marker occurred particularly within the first hour, which coincides with the procedural time for, e.g., a stent implantation procedure. Future experiments should include metrics such as viscosity and molecular weight to characterize the synthesized PLA in more detail and thus allow further modulation of its degradation characteristics. To exclude potential magnetic effects, we chose to work with glass capillaries as carriers for the PLA‐SPION mixture. In future studies, the marking technology should be applied and tested on different implants, e.g., stents, and coils. As PLA degradation is multifactorial, and in vivo, testing is essential for potential medical translation of these initial results.

## 5 Conclusion

This work introduces a PLA‐based MPI marking technology. Especially, the combination of significant PLA mass loss and a constantly detectable MPI signal is the basis for the functionality of the presented technique in anticipated medical use cases. Despite the need for further experimental work, the application scenario is promising and an important accompanying step for the pending clinical introduction of MPI.

## Conflict of Interest

The authors declare no conflict of interest.

## Data Availability

The data that support the findings of this study are available from the corresponding author upon reasonable request.
